# Mutation Analysis of Second Primary Tumors in Oral Cancer in Taiwanese Patients through Next-Generation Sequencing

**DOI:** 10.3390/diagnostics12040951

**Published:** 2022-04-11

**Authors:** Ting-Yuan Liu, Chien-Chin Lee, Yu-Chia Chen, Ya-Sian Chang, Hsi-Yuan Huang, Ya-Ting Lee, Ju-Chen Yen, Dysan Chao, Jan-Gowth Chang

**Affiliations:** 1 Center for Precision Medicine, China Medical University Hospital, Taichung 40447, Taiwan; t92990@mail.cmuh.org.tw (T.-Y.L.); t92989@mail.cmuh.org.tw (Y.-C.C.); t25074@mail.cmuh.org.tw (Y.-S.C.); t31766@mail.cmuh.org.tw (D.C.); 2 Epigenome Research Center, China Medical University Hospital, Taichung 40447, Taiwan; insect@hotmail.com.tw (C.-C.L.); t23701@mail.cmuh.org.tw (Y.-T.L.); t24399@mail.cmuh.org.tw (J.-C.Y.); 3 School of Life and Health Sciences, The Chinese University of Hong Kong, Shenzhen 518172, China; cn0312@gmail.com; 4 Warshel Institute for Computational Biology, The Chinese University of Hong Kong, Longgang District, Shenzhen 518172, China

**Keywords:** oral cancer, second primary tumors, next-generation sequencing, driver gene, trunk mutations

## Abstract

Head and neck cancer has poor overall survival. Patients with head and neck cancer more frequently develop second primary tumors than do patients with other cancers, leading to a poor prognosis. In this study, we used next-generation sequencing to analyze and compare mutations between first tumors and second tumors in oral cancer. We retrieved tumor tissues collected from 13 patients who were diagnosed twice as having cancer. We used driver gene and trunk mutations to distinguish between recurrent cancer and primary cancer in oral cancer. We observed unique driver gene mutations in three patients with an initial clinical diagnosis of recurrent cancer; hence, we believe that the corresponding patients had primary cancer. Four patients with an initial clinical diagnosis of primary cancer were found to actually have recurrent cancer according to our results. Genetic testing can be used to enhance the accuracy of clinical diagnosis.

## 1. Introduction

Head and neck cancers (HNCs) constitute a group of cancers that occur in the mouth, nose, throat, larynx, sinuses, or salivary glands. The symptoms of HNC vary depending on the cancer type [1], with some patients presenting with a nonhealing lump or sore in the mouth and others presenting with a persistent sore throat. Other patients experience trouble swallowing or a change in voice [2]. Nearly 75% of HNCs are caused by the use of alcohol or tobacco [3,4]. However, in Taiwan, such cancers are often caused by areca. Studies have revealed that areca is an essential risk factor for HNC development [5,6]. This trend is different from those reported in Western countries because genomic alterations in HNC differ between the West and the East.

HNC is considered the sixth most common cancer worldwide and constitutes 6% and 3% of cancer-related deaths in men and women, respectively [7,8]. In Taiwan, approximately 7000 people are diagnosed as having HNC, and this cancer causes more than 3000 deaths each year (https://www.hpa.gov.tw/, 24 December 2019). A critical reason for the poor overall survival is that patients with HNC more frequently develop second primary tumors (SPTs) than do patients with other cancers, leading to poor prognosis [9,10]. They are defined as second tumors (STs) that manifest either simultaneously or after the diagnosis of the first tumor (FT). SPTs must be differentiated from local recurrences or primary tumor metastases [11]. Patients with HNC have a high lifetime risk of developing SPTs; the incidence of SPTs in such patients is 2% to 3% annually [8,10,12,13]. SPT diagnostic criteria were first presented by Warren and Gates in 1932; in these criteria, an SPT is defined as a new malignant tumor that is located at a new anatomic side and is adequately separated from the original lesion [14]. On the basis of recent molecular analysis results, the SPT criteria have been modified; the modified criteria suggest that individual tumors arising in the same field as premalignant lesions with different genomic alterations might be regarded as SPTs.

To explain the development of multiple primary tumors in HNC, Slaughter et al. proposed the concept of “field cancerization,” which indicates that, when large areas of mucosa are exposed to carcinogens for a prolonged period, a variety of precancerous lesions are formed, which eventually develop into several independent primary tumors [15]. These events involve multistep processes, including genetic alterations; damage induced by carcinogens, such as tobacco and alcohol; and human papillomavirus (HPV) infection. The discovery of genetic changes appears to support this concept of the origin of independent tumors [16,17]. SPTs constitute the second leading cause of death in patients with HNC [18]. In this study, we identified mutant verifications as markers of SPTs.

HNCs are highly related to lifestyle risk factors, and different forms and levels of exposure to the etiological agents are reflected in different parts of the world. Studies have reported that cancer development involves the accumulation of mutations in oncogenes or tumor suppression genes [19,20,21]; most of such studies have focused on tobacco- and alcohol-related HNCs and rarely on betel quid (BQ)-related HNC. To address this gap in the literature, we collected cancer tissues from 15 patients with SPTs and used next-generation sequencing (NGS) to analyze the mutations in first primary tumors (FPTs) and SPTs to explore the possible signaling pathways between them.

## 2. Materials and Methods

### 2.1. Patients and Samples

Tumor samples collected from 13 patients with SPT were retrieved from the human biobank of China Medical University Hospital, Taiwan. DNA was extracted using the QIAamp DNA Micro kit (Qiagen, Heidelberg, Germany) according to the manufacturer’s protocol. The extracted DNA samples were then quantified using a NanoDrop 2000 spectrophotometer (Thermal Fisher Scientific, Waltham, MA, USA) and Qubit fluorometer (Invitrogen, Carlsbad, CA, USA). The Institutional Review Board of China Medical University Hospital (CMUH102-REC1-015, 13 March 2013 approved and CMUH102-REC1-073, 23 September 2013 approved) approved our study.

### 2.2. Exome Capture and Massively Parallel Sequencing

A TruSeq DNA Sample Preparation Kit (Illumina, San Diego, CA, USA) was used to create the DNA library in accordance with the manufacturer’s guidelines. Genomic DNA (5 μg) was fragmented using a Covaris sonicator (Covaris, Woburn, MA, USA) to a size of 300–500 bps. The library preparation process involved the following steps: enzyme-mediated end repair, adenine addition a-tailing, adapter oligonucleotide ligation, and adapter-ligated fragment enrichment through a limited-cycle polymerase chain reaction (PCR). Human exome capture was performed according to the Illumina TruSeq Exome Enrichment Kit protocol. The DNA library was subjected to denaturation at 95 °C for 10 min and was subjected to hybridization at 58 °C for 16 h using captured probes. Subsequently, streptavidin beads were used to bind biotin-labeled probes that contained the targeted regions of interest. Three washing steps were performed to inhibit nonspecific binding to the beads. The hybridization and washing steps were then repeated. Next, PCR was executed to amplify the enriched DNA library for sequencing, after which the enriched DNA library was purified using an AMPure XP purification system (Agencourt, Beckman Coulter, Brea, CA, USA). Final quantification of the libraries was performed using a Qubit 2.0 Fluorometer high-sensitivity DNA assay (Invitrogen) and an Experion Automated Electrophoresis System (Bio-Rad, Hercules, CA, USA) to ensure sufficient product availability for sample normalization and pooling. Library-prepared samples were sequenced using a HiSeq platform (Illumina, San Diego, CA, USA) to produce 100-bps paired-end sequencing reads.

### 2.3. Data Analysis

We performed base calling and quality scoring using an updated implementation of real-time analysis on the aforementioned HiSeq platform. Data were demultiplexed, and BCL files were converted to FASTQ files through Bcl2fastq conversion software. Subsequently, the sequenced reads for low-quality sequences were trimmed, after which they were aligned to the human reference genome (hg19) using the Burrows-Wheeler Alignment tool [22]. Small insertions, deletions, or both, and single-nucleotide polymorphisms (SNPs) were next identified in each sample through the Genome Analysis Toolkit and VarScan under their default settings [23,24]. We then applied ANNOVAR [25] and household script to perform gene-based, region-based, and filter-based annotation to functionally annotate variants. Finally, the variants were annotated using several databases and tools, including dbSNP (build 147), ClinVar, COSMIC (ver. 70), TCGA, Polyphen-2, SIFT, and CADD [20,21,22,23,24,25,26].

### 2.4. Variant Validation through Sanger Sequencing

Primer3 software (Supplementary Table S1) was used to design the PCR primers in silico. We used a Verity 96-well thermal cycler (Applied Biosystems, Foster City, CA, USA) to perform PCR including specific primers, after which we executed conventional PCR-based Sanger sequencing using an ABI 3130 DNA analyzer (Applied Biosystems).

## 3. Results

### 3.1. Population Description and Clinical Information in SPT

We retrieved tumor samples collected from 13 patients with SPTs. Of these patients, 12 had FPTs and SPTs in HNCs (Table 1), and one had an FPT in HNC and an SPT in the esophagus, and one had an FPT in the ureter and an SPT in HNC. Ten (77%) patients had habits of smoking, BQ chewing, and drinking; two had habits of BQ chewing and drinking, and one (7%) had none of the aforementioned habits. Table 1 presents the clinical features of SPTs. All cancers were of the squamous cell carcinoma (SSC) type. The pathological tumor–node–metastasis (pTNM) classification system was established by the American Joint Committee on Cancer (AJCC) and the International Union against Cancer to avoid heterogeneity in prognostic classification schemes used for differentiated cancers. The AJCC has created a set of resource materials that provide in-depth information to medical professionals and cancer registrars for staging cancer patients and abstracting cancer cases, respectively. In this study, we used the pTNM classification system to classify tumors and the AJCC staging system to determine tumor stages and evaluate tumor size; the results are presented in Table 1. We divided patients into two groups (Table 1): the upper group, comprising patients clinically diagnosed twice as having primary cancer; and bottom group, comprising patients clinically diagnosed as having recurrent cancers; We recorded the treatment policy for each cancer, including chemotherapy (CT) and radiation therapy (RT).

### 3.2. Identifying Variants in SPTs

We performed massive parallel sequencing by using the HiSeq platform. We generated nearly 160 M raw reads per sample, on average; these reads were aligned with the human reference genome (hg19; Supplementary Table S2). The target regions of the 26 samples exhibited a mean depth and coverage of 141 (range: 92.37–175.21) and 99.19% (range: 98.95–99.35%), respectively. Supplementary Figure S1 illustrates a schematic of our variant identification approach. We executed whole-exon sequencing to collect data of variants from our patients’ DNA. The ratio of variant reads to total reads must be greater than 10%. The ratio of variant reads less than 10% may be mistake by amplification or NGS. Subsequently, we used the dbSNP and genome-wide association study (GWAS) database to annotate variants with global minor allele frequencies of more than 1%. Moreover, we used the ClinVar database to annotate the remaining variants. Variants were divided into the following categories: pathogenic, benign, and uncertain. Benign variants were annotated using the dbSNP, COSMIC, and HGVS databases. The pathogenicity of uncertain variants was predicted using the SIFT, PolyPhen, and Combined Annotation Dependent Depletion (CADD) tools.

### 3.3. Cancer-Related Gene Mutational Status in HNC

**Figure 1:**
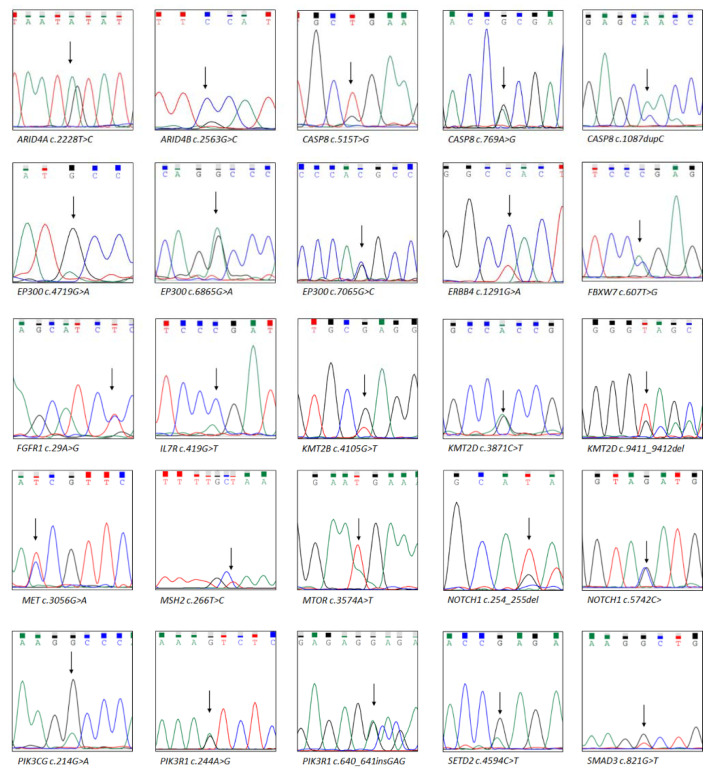
Driver gene mutations confirmed using Sanger sequencing. Next-generation sequencing data provided these driver gene mutations. The arrow indicates the location of the mutation.

This study included 756 canonical cancer-related genes. We detected 297 mutations in 190 of these genes, namely seven frameshift deletions, three frameshift insertions, 261 missense mutations, five non-frameshift deletions, two non-frameshift insertions, and 19 stop-gains. *SYNE1* (56%; 15/27), *TP53* (52%; 14/27), and *CDKN2A* (41%; 11/27) were the most frequently mutated genes in HNC. A total of 215 variants were identified in the COSMIC, dbSNP, and TCGA databases, but 75 variants in 80 genes were not identified in these databases (Supplementary Table S1). We executed Sanger sequencing to verify these driver gene variants (Figure 1) and nondriver gene variants (Figure S2).

### 3.4. Mutations Analysis in FPT, SPT, and Intersection Parts

**Figure 2:**
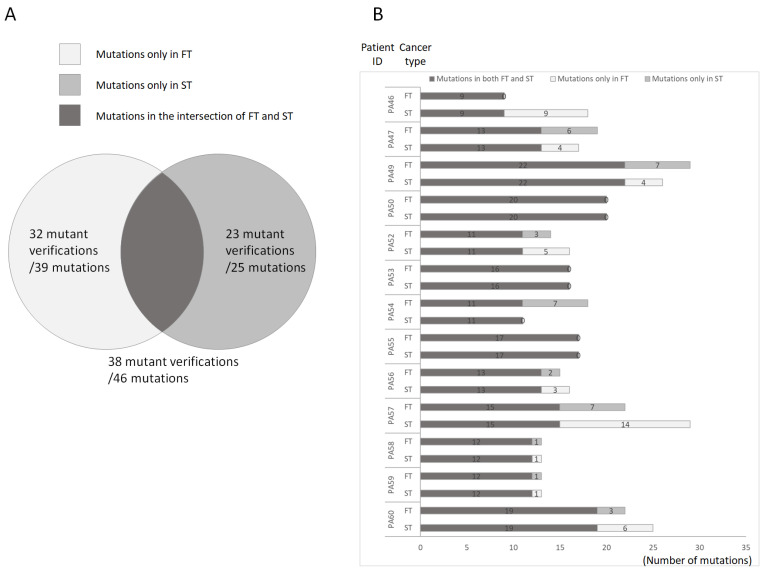
Schematic of the distribution of mutations in first and second primary tumors. (**A**). Overview of mutations in first and second tumors. Only first primary tumor showed 39 mutations (White). We verified 32 of the 39 mutations. Only second primary tumor showed 25 mutations (gray). We verified 23 of the 25 mutations. Both first and second primary tumors showed 46 mutations (black). We verified 38 of the 46 mutations. (**B**). Distribution of mutations in patients. This bar chart shows mutations in first primary tumor (white), second primary tumor (gray), and first and second primary tumors (black). The number in the bar shows the number of mutations.

To explore differences in mutations between the FT and the ST in each patient, we divided the observed mutations into three categories: those found only in the FT (oFTp), those found only in the ST (oSTp), and those found in the intersection between these tumors (Figure 2A). NGS revealed nine identical mutations in PA46 patients and nine unique mutations in oSTp mutations. In PA50, PA53, and PA55, the mutations in the FT and ST were the same; PA54 and oFTp had 11 identical and seven unique mutations, respectively; in PA47, PA49, PA52, PA56, PA57, PA58, PA59, and PA60, the mutations in the FT and ST were different (Figure 2B). These classification theories can thus be used to distinguish a second primary oral cancer from other cancers. 

### 3.5. Molecular Diagnosis according to Driver Gene Mutations for Differentiating between the FT and ST

**Figure 3:**
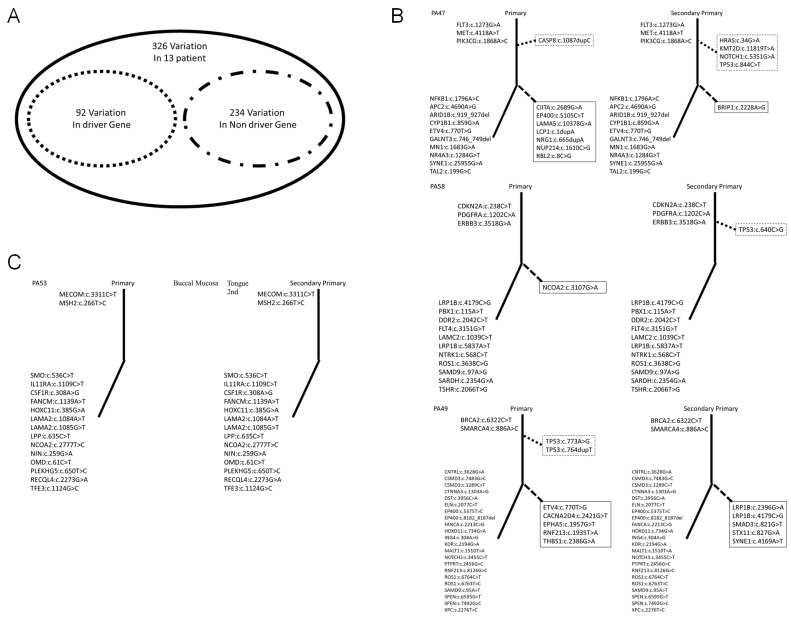
Analysis of driver gene and trunk mutations in first and second primary tumors. (**A**). For the 15 patients, we observed 297 mutations. The corresponding genes could be divided into driver genes (83 genes) and nondriver genes (214 genes). (**B**). First and second primary tumors as categorized according to our results. The solid and dotted lines show trunk and branch mutations, respectively. The upper and lower parts of the graph show driver and nondriver gene mutations, respectively. The dotted line box shows driver gene mutations and trunk mutations. The solid line box shows nondriver gene mutations and branch mutations. (**C**). Recurrent cancers as categorized in this study.

As revealed by the results in the preceding section, we explored differences in mutations between the FT and ST in each patient. We also determined each patient’s clinical diagnosis, as presented in Table 1. For further exploration, we used molecular diagnoses made according to driver gene mutations to distinguish between the FT and ST in each patient. Driver genes are necessary for cancer to become malignant. We used 299 driver cancer genes to distinguish between the FT and ST [26,27]. Cancer is a microevolutionary process that originates from a single cell [28,29,30]. The classification of trunk and branch mutations can elucidate the microevolution of cancer [31]. Therefore, we distinguished between trunk and branch mutations in each cancer. A total of 297 genes with verified mutations were classified into driver and nondriver categories (Figure S3). Of the, 83 and 214 were driver and nondriver genes, respectively (Figure 3A). We derived representative results of trunk and branch mutations in primary (Figure 3B) and recurrent (Figure 3C) cancers. We present representative patients in Figure 3 and the remaining results are presented in Figure S4. We sorted the unique mutations in each cancer (Table 2). As mentioned, we divided patients into upper, and bottom groups according to clinical diagnosis. The upper group comprised nine patients. Nevertheless, we believe that three of them (PA50, PA53, and PA55, 3/9) had recurrent cancer because we did not observe a unique driver gene mutation in the ST. The bottom group comprised four patients. However, we observed unique driver gene mutations in PA52, PA57, and PA59; hence, we believe that the corresponding patients had primary cancer.

## 4. Discussion

In this study, we retrieved tissue samples collected from 15 patients who were diagnosed twice as having cancer. We used whole exome sequencing to analyze genetic changes in these cancers. Furthermore, we verified these driver gene variants and applied molecular diagnosis according to driver gene and trunk mutations in recurrent cancer to distinguish between first and recurrent cancers.

In clinical practice, cancer recurrence is diagnosed according to physicians’ judgment and pathological biopsy findings. In the literature, recurrence is defined as a reemergence of cancer at the same location or a nearby site within a short interval after the first diagnosis. It is also defined as pathological biopsy findings revealing the same morphology and malignancy for both occurrences of cancer [32]. The aim of the present study was to describe the differences in mutations between the FT and ST in cancer and evaluate whether the ST is an SPT or recurrence of the FT using molecular diagnosis. Liu conducted a molecular diagnosis for recurrent cancer according to driver gene and trunk mutations [33]. Our results reveal that some patients who were initially diagnosed as having primary cancer actually had recurrent cancer. These recurrent cancers did not show unique driver gene mutations (Table 2). Driver gene and trunk mutations may become a new diagnostic biomarker for distinguishing between recurrent cancer and primary cancer.

In our study, four patients were determined to have recurrent cancer according to our results (Table 2). In one patient (PA53), we did not observe any unique gene mutation between both occurrences of cancer; therefore, the cancer was clearly recurrent. Two patients (PA50, and PA55) showed unique nondriver gene mutations in the second diagnosis of cancer. These patients had undergone either CT or RT after the first cancer diagnosed (Table 1). There may be two groups of cancer cells in such patients, with part of the cancer cells dying after the treatment [34]. These patients also had recurrent cancer. Notably, for the four patients who were clinically diagnosed as having recurrent cancer, our results indicated that they had primary cancer. This result shows the inaccuracy of clinical interpretation.

We analyzed 13 patients who were diagnosed twice as having cancer. Using driver gene and trunk mutations, we distinguished between recurrent and primary cancer oral cancers. These findings may require further research for confirmation.

## Figures and Tables

**Table 1:** Description and clinical information of patients included in the study.

ID	Gender	Betel	Alcohol	Smoking	Sample ID	Age at Surgery	Interval Time (Days)	Organization	Pathol. Diagnosis	Tumor size (cm)	pTNM	Stage (AJCC)	Clinical Diagnosis	CT	RT
PA46	M	+	+	−	PM245	64		Right Buccal Mucosa	SSC	4.0 × 2.0 × 0.4	pT2N2bcMx	IVA	1st primary	+	+
					PM558	68	1216	Lip	SSC	3.2 × 3.2 × 0.8	ypT2N0cMx	IVA	2nd primary	−	−
PA47	M	−	−	−	PM247	58		Gum	SSC	13 × 5.5 × 6.5	pT3N0cM0	III	2nd primary	−	−
					PM571	62	1364	Gum	SSC	2.8 × 2.2 × 0.4	pT2N0cMx	II	3rd primary	−	−
PA49	M	+	+	+	PM251	45		Larynx	SSC	3.5 × 3.5 × 3.0	pT4aN2cM0	IVA	1st primary	+	+
					PM560	51	2236	Gum	SSC	3.0 × 2.5 × 1.5	ypT4aNxcM0	IVA	2nd primary	+	+
PA50	M	+	+	−	PM253	71		Gum	SSC	0.6 × 0.6 × 0.2	pT4aN0cM0	IVA	1st primary	−	+
					PM561	75	1716	Gum	SSC	2.7 × 1.8 × 1.1	ypT4aNxcM0	IVA	2nd primary	+	+
PA53	M	+	+	+	PM259	40		Buccal Mucosa	SSC	2.3 × 1.8 × 0.4	pT2N2bcMx	IVA	1st primary	+	+
					PM563	42	728	Tongue	SSC	1.0 × 0.8 × 0.5	ypT1NxcM0	I	2nd primary	−	−
PA54	M	+	+	+	PM573	51		Buccal Mucosa	SSC	4.0 × 3.0 × 2.5	pT4aN0cM0	IVA	1st primary	−	+
					PM261	57	2415	Hypopharynx	SSC	3.1 × 3.1 × 0.7	ypT2N0cM0	II	2nd primary	−	−
PA55	M	+	+	+	PM265	50		Gum	SSC	4.0 × 3.5 × 1.3	pT4aN2bcM0	IVA	1st primary	+	+
					PM564	55	2001	Gum	SSC	2.4 × 1.5 × 1.5	pT4aN0M0	IVA	3rd primary	+	+
PA58	M	+	+	+	PM276	45		Buccal Mucosa	SSC	2.5 × 2.5 × 0.6	pT2N0cMx	II	1st primary	−	−
					PM568	49	1544	Gum	SSC	3.0 × 1.8 × 1.0	pT4aN0cM0	IVA	3rd primary	+	+
PA60	M	+	+	+	PM281	46		Tongue	SSC	3.3 × 2.8 × 2.0	pT2N2bcMx	IVA	1st primary	+	+
					PM570	46	341	Esophagus	SSC	5.0 × 3.0 × 1.5	ypT4N1cMx	III	2nd primary	+	+
PA52	M	+	+	+	PM257	65		Tongue	SSC	1.7 × 1.7 × 1.0	pT1N0cMx	I	1st primary	-	-
					PM562	66	386	Tongue	SSC	4.3 × 4.1 × 1.4	rpT3N2bcM0	IVA	recurrent	+	+
PA56	M	+	+	+	PM267	56		Gum	SSC	3.0 × 1.3 × 1.3	pT2N0cM0	II	1st primary	−	−
					PM565	65	3492	Lip	SSC	2.5 × 2.3 × 0.5	rpT1NxcM0	I	recurrent	−	−
PA57	M	+	+	+	PM269	51		Gum	SSC	3.0 × 2.0 × 2.0	pT4aN0cM0	IVA	1st primary	−	−
					PM566	57	2436	Buccal Mucosa	SSC	5.3 × 5.3 × 1.4	rpT3NxM0	III	recurrent	+	+
PA59	M	+	+	+	PM569	47		Oropharynx	SSC	4.2 × 4.2 × 3.5	pT4N1cM0	IVA	1st primary	+	+
					PM279	55	2881	Buccal Mucosa	SSC	3.3 × 2.2 × 1.5	yrpT4aN0cM0	IVA	recurrent	+	+

F: female, M: male, SSC: squamous cell carcinoma, TCC: transitional cell carcinoma; AJCC: the American Joint Committee on Cancer; CT: chemotherapy; RT: radiation therapy; pTNM: pathological tumor–node–metastasis. T: size of the original tumor and whether it has invaded nearby tissue. N: nearby lymph nodes that are involved. M: distant metastasis. c: stage determined from evidence acquired before treatment. p: derived through histopathological examination of a surgical specimen. y: stage assessed after chemotherapy and/or radiation therapy. r: stage of recurrent tumor in an individual. +: positive. −: negative.

**Table 2:** Unique mutations in first and second primary tumors.

ID	Sample ID	Organization	Clinical Diagnosis	Unique Mutation	Molecular Identification
PA46	PM245	Buccal Mucosa	1st primary	N/A	
	PM558	Lip	2nd primary	CASP8:c.515T>G; MLH1:c.2260G>A; NOTCH1:c.5742C>G; PIK3CG:c.214G>A; SETD2:c.4594C>T; ADAMTS20:c.1066C>T; FLCN:c.502C>T; NCOA2:c.2197G>A; SYNE1:c.21119G>A	primary
PA47	PM247	Gum	2nd primary	CASP8:c.1087dupC; CIITA:c.2689G>A; EP400:c.5105C>T; LAMA5:c.10378G>A; NRG1:c.665dupA; NUP214:c.1610C>G	
	PM571	Gum	3rd primary	HRAS:c.34G>A; NOTCH1:c.5351G>A; TP53:c.844C>T; BRIP1:c.2228A>G	primary
PA49	PM251	Larynx	1st primary	TP53:c.773A>G; ETV4:c.770T>G; CACNA2D4:c.2421G>T; EPHA5:c.1957G>T; RNF213:c.1935T>A; THBS1:c.2386G>A	
	PM560	Gum	2nd primary	LRP1B:c.2396G>A; SMAD3:c.821G>T; STX11:c.827G>A; SYNE1:c.4169A>T	primary
PA50	PM253	Gum	1st primary	N/A	
	PM561	Gum	2nd primary	N/A	recurrent
PA53	PM259	Buccal Mucosa	1st primary	N/A	
	PM563	Tongue	2nd primary	N/A	recurrent
PA54	PM573	Buccal Mucosa	1st primary	CASP8:c.305G>A; FBXW7:c.607T>G; MET:c.3056G>A; NOTCH1:c.1057C>T; TGFBR2:c.1651G>A; ADAMTS20:c.3598G>T	
	PM261	Hypopharynx	2nd primary	N/A	primary
PA55	PM265	Gum	1st primary	N/A	
	PM564	Gum	3rd primary	N/A	recurrent
PA58	PM276	Buccal Mucosa	1st primary	NCOA2:c.3107G>A	
	PM568	Gum	3rd primary	TP53:c.640C>G	primary
PA60	PM281	Tongue	1st primary	AKT1:c.1133C>A; ATRX:c.830T>C; ARID4A:c.2228T>C	
	PM570	Esophagus	2nd primary	APC:c.4315C>T; IL7R:c.419G>T; TP53:c.781delA; PPARG:c.1276C>A; PRDM16:c.1442C>G	primary
PA52	PM257	Tongue	1st primary	HRAS:c.34G>A; BRD4:c.3845G>A	
	PM562	Tongue	recurrent	CDKN2A:c.238C>T; TP53:c.527G>T; HECW1:c.2872C>T; TAL1:c.503A>C; UBR5:c.6859G>T	primary
PA56	PM267	Gum	1st primary	ERBB4:c.1291G>A; PIK3R1:c.640_641insGAG	
	PM565	Lip	recurrent	APC2:c.4690A>G; DST:c.13567C>T; ZNF521:c.2773G>A	primary
PA57	PM269	Gum	1st primary	CASP8:c.652C>T; FBXW7:c.1273A>G; SMARCA4:c.4285C>T; TP53:c.772G>A; EPS15:c.76G>A; FANCM:c.3257G>A; MTR:c.3482C>A	
	PM566	Buccal Mucosa	recurrent	CDKN2A:c.238C>T; CASP8:c.319C>T; ERCC2:c.860G>A; HRAS:c.182A>T; NOTCH1:c.254_255del; PBRM1:c.1028C>G; SF3B1:c.1390C>T; ALDH2:c.1303G>T; ARID4B:c.2563G>C; EPHA5:c.1582G>T; GUCY1A2:c.463C>T; RAPGEF2:c.4430G>T; SYNE1:c.6035G>A; TSHR:c.2063A>C	primary
PA59	PM569	Oropharynx	1st primary	LTK:c.638_639insTGGCGGGGG; PKHD1:c.1282delT	
	PM279	Buccal Mucosa	recurrent	CDKN2A:c.220G>T	primary

## Data Availability

The data used in this paper is in Supplementary Information.

## Supplementary Materials

The following supporting information can be downloaded at: https://www.mdpi.com/article/10.3390/diagnostics12040951/s1, Figure S1: Overview of our approach to identifying variants in SPTs; Figure S2: Overview of our approach to identifying variants in SPTs; Figure S3: A total of 297 genes with verified mutations were classified into driver and nondriver categories; Figure S4: Analysis of driver gene and trunk mutations in first and second primary tumors; Table S1. Primer list; Table S2. Overview of reads count and quality control from NGS; Table S3. Overview of 297 verified mutations; Data S1: OralCancer_Raw_Data.

## Abbreviations

American Joint Committee on Cancer	AJCC
betel quid	BQ
chemotherapy	CT
Combined Annotation Dependent Depletion	CADD
first primary tumors	FPTs
first tumor	FT
genome-wide association study	GWAS
Head and neck cancers	HNCs
human papillomavirus	HPV
human reference genome	hg19
next-generation sequencing	NGS
only in the FT	oFTp
only in the ST	oSTp
pathological tumor–node–metastasis	pTNM
polymerase chain reaction	PCR
radiation therapy	RT
second primary tumors	SPTs
Second tumors	STs
single-nucleotide polymorphisms	SNPs
squamous cell carcinoma	SSC
transitional cell carcinoma	TCC
