# Supplementary material for: Mutation Analysis of Second Primary Tumors in Oral Cancer in Taiwanese Patients through Next-Generation Sequencing
Source: Diagnostics (Basel). 2022 Apr 11;12(4):951. doi: 10.3390/diagnostics12040951 (PMC9025858; doi:10.3390/diagnostics12040951)
Supplement: Supplementary file 1 [file diagnostics-12-00951-s001.zip › diagnostics-1583091-supplementary.pdf]

Figure S1

Overview of our approach to identifying variants in SPTs

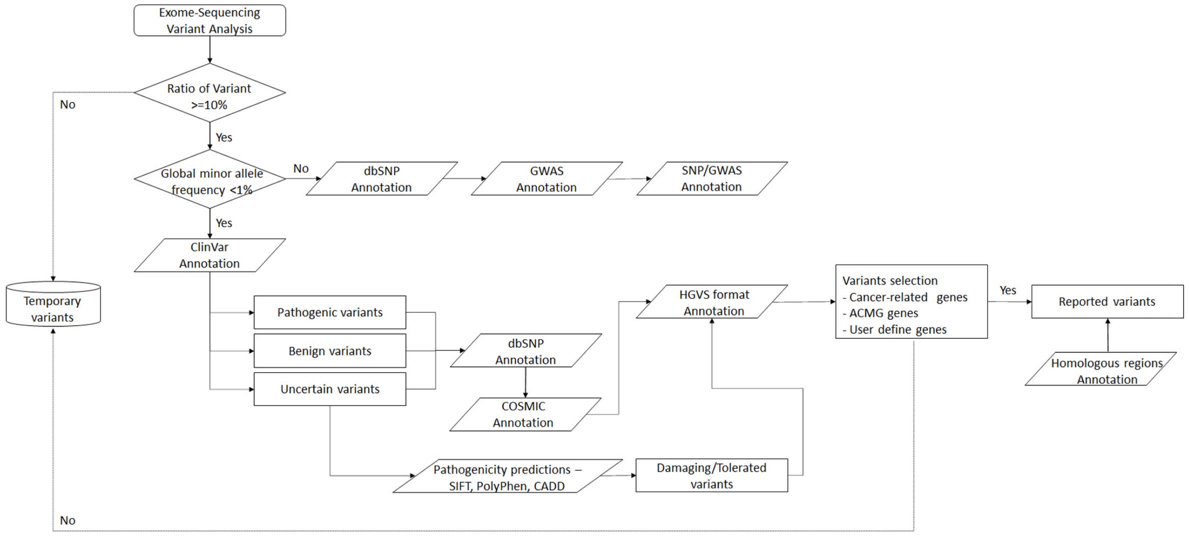

Figure S1. Overview of our approach to identifying variants in SPTs.

Figure S2

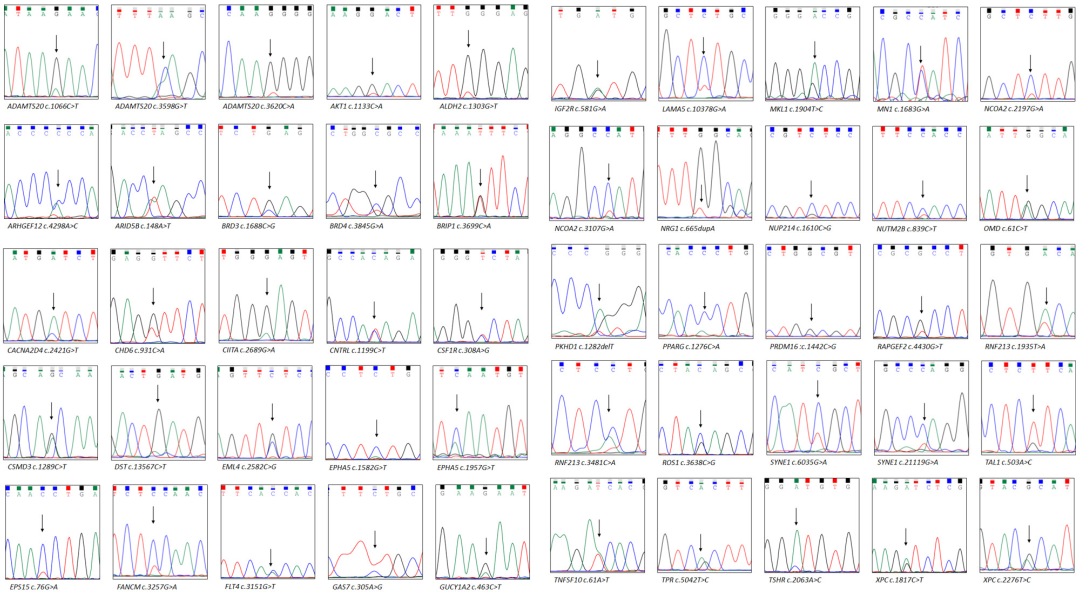

Figure S2. Overview of our approach to identifying variants in SPTs.

Fig S3

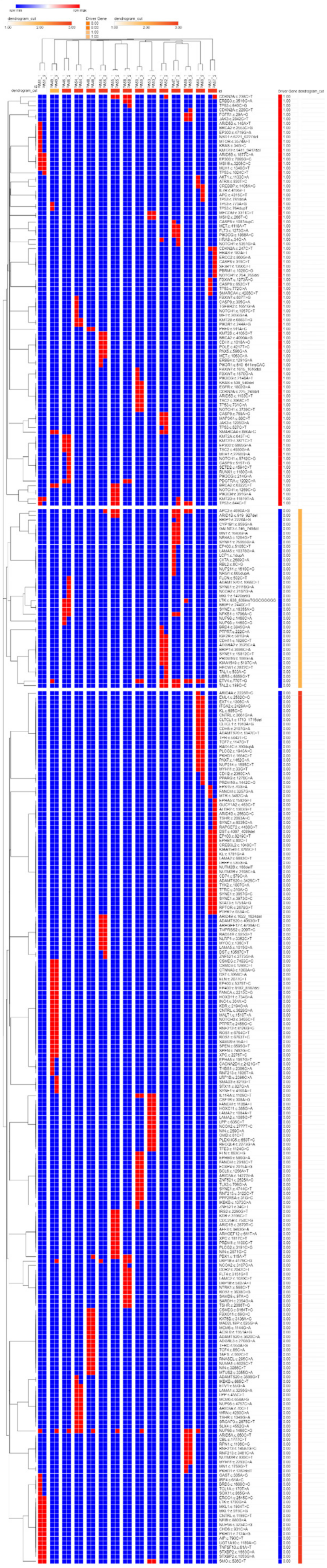

Figure S3. A total of 297 genes with verified mutations were classified into driver and nondriver categories.

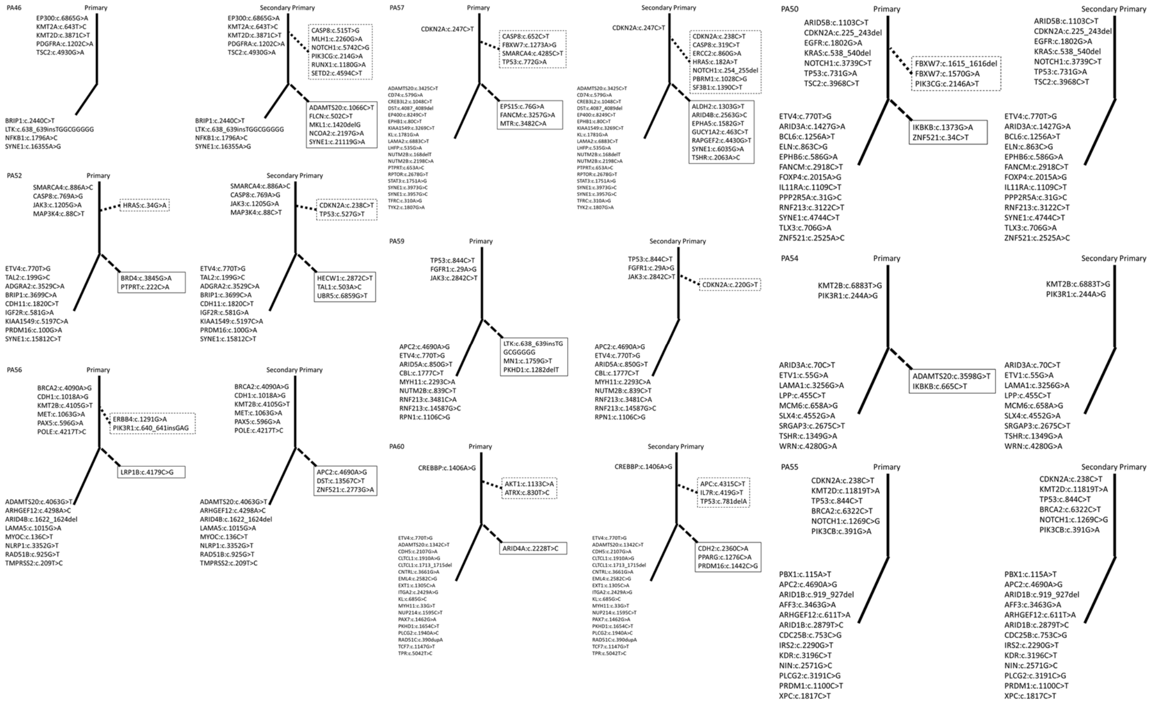

Figure S4. Analysis of driver gene and trunk mutations in first and second primary tumors.
